# Association between triglyceride-to-high-density lipoprotein cholesterol ratio and albuminuria in patients with type 2 diabetes: a cross-sectional study

**DOI:** 10.3389/fnut.2026.1810515

**Published:** 2026-07-01

**Authors:** Jinghan Zheng, Jingwei Chi, Kui Che, Yangang Wang, Qidong Zheng

**Affiliations:** 1 Department of Endocrinology, Affiliated Hospital of Qingdao University, Qingdao, Shandong, China; 2 Department of Internal Medicine, Yuhuan Second People's Hospital, Yuhuan, Zhejiang, China

**Keywords:** albuminuria, insulin resistance, non-linear association, TG/HDL-C, type 2 diabetes

## Abstract

**Background:**

Previous studies have demonstrated that the triglyceride (TG) to high-density lipoprotein cholesterol (HDL-C) ratio is closely related to chronic kidney disease and albuminuria. However, the evidence linking TG/HDL-C ratios to albuminuria among individuals with type 2 diabetes (T2D) remains limited. Given the heightened susceptibility of this high-risk population to kidney and cardiovascular ailments, this study concentrated on individuals with T2D to investigate the association between TG/HDL-C and albuminuria.

**Methods:**

A total of 2,323 people diagnosed with T2D participated in this cross-sectional research. The relationship between TG/HDL-C and albuminuria prevalence was evaluated using logistic regression analysis. Restricted cubic spline (RCS) models were applied to identify nonlinear relationships. The ability of TG/HDL-C versus other markers to distinguish albuminuria was examined using receiver operating characteristic (ROC) curves. Further subgroup analyses were conducted to explore heterogeneity across different populations. In addition, we explored whether serum uric acid (SUA) and fasting blood glucose (FBG) accounted for part of the association between TG/HDL-C and albuminuria.

**Results:**

The albuminuria group and the non-albuminuria group showed a significant difference in TG/HDL-C. Additionally, the prevalence of albuminuria increased progressively across ascending quartiles of TG/HDL-C. RCS curve analysis revealed that albuminuria and TG/HDL-C do not follow a linear relationship (P-overall < 0.001; P-nonlinear < 0.001), with a threshold observed at a TG/HDL-C ratio of 2.08. Furthermore, TG/HDL-C demonstrated relatively higher discrimination, particularly in women. Subgroup analysis indicated that among female T2D patients, TG/HDL-C was more strongly linked to UACR. Exploratory mediation analysis suggested that blood glucose and oxidative stress may contribute to the indirect pathway linking TG/HDL-C and albuminuria.

**Conclusion:**

In conclusion, the TG/HDL-C ratio was positively associated with the prevalence of albuminuria in patients with T2D. However, causal inference is limited by the cross-sectional design. Further prospective studies are needed to confirm our findings.

## 1 Introduction

Type 2 diabetes (T2D), a chronic metabolic disorder marked by hyperglycemia, is witnessing a progressive increase in worldwide prevalence, representing a major public health concern (1). According to data from the International Diabetes Federation (IDF), the global prevalence of diabetes in 2021 was estimated at 10.5%, projected to reach 12.2% by 2045 (2), with T2D accounting for approximately 90% of cases (3). The presence of albuminuria — marked by increased urinary albumin excretion — constitutes an early sign of renal injury (4). It is reported that albuminuria is present in up to 40% of diabetic patients (5) and represents an independent risk factor for cardiovascular and renal events in individuals with T2D (6).

Insulin resistance and atherogenic dyslipidemia are central pathophysiological features of T2D. The triglyceride-to-high-density lipoprotein cholesterol (TG/HDL-C) ratio serves as a readily available surrogate marker of these metabolic disturbances. Elevated TG/HDL-C reflects increased hepatic secretion of triglyceride-rich lipoproteins and impaired reverse cholesterol transport, fostering lipid accumulation in non-adipose tissues and promoting systemic inflammation (7). In the context of diabetes, these lipid abnormalities may exacerbate pancreatic β-cell dysfunction and glucolipotoxicity, worsening glycemic control. Beyond glycemic deterioration, dyslipidemia contributes to end-organ damage through oxidative stress and pro-inflammatory cytokine activation, processes that may compromise glomerular filtration barrier integrity and increase albumin permeability — thereby linking TG/HDL-C to albuminuria through both direct lipotoxic effects on renal parenchyma and indirect mediation via insulin resistance-related pathways (8).

Epidemiological studies have supported these biological plausibilities. Prior investigations have established a close association between TG/HDL-C and insulin resistance (9), as well as central obesity (10). Furthermore, TG/HDL-C is considered a novel predictor of metabolic syndrome, cardiovascular diseases (11, 12), and chronic kidney disease—all of which are closely linked to albuminuria. Several studies have demonstrated a positive correlation between TG/HDL-C and the UACR (13, 14). However, investigations into the relationship between TG/HDL-C and proteinuria in diabetic patients remain relatively scarce. Given that this high-risk group is particularly susceptible to renal and cardiovascular diseases, we conducted a cross-sectional study among T2D patients across two research centers to explore the association between TG/HDL-C and albuminuria.

## 2 Methods

### 2.1 Study population

**Figure 1:**
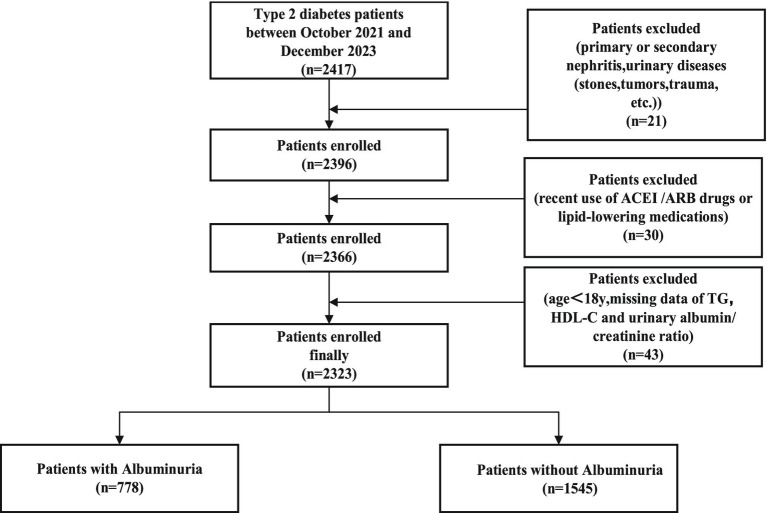
Flowchart of the study.

This research was a cross-sectional analysis of two different populations. Using the electronic medical data of the Affiliated Hospital of Medical College Qingdao University and the National Metabolic Management Centre (MMC) (15) of Yuhuan Second People’s Hospital, a total of 2,495 diabetic subjects were recruited from October 2021 to December 2023. The MMC is a nationwide initiative that provides standardized, one-stop medical services for patients with diabetes and other metabolic diseases under the guiding principle of “one center, one-stop service, one standard”. The network enforces consistent clinical protocols and quality standards throughout its tiered infrastructure. During recruitment, all participant data were systematically gathered by locally trained MMC staff following rigorously standardized procedures, thereby maintaining uniformity in demographic, clinical, and laboratory variables. The inclusion criterion was individuals meeting the 2022 American Diabetes Association’s diagnostic criteria of T2D (16). Exclusion criteria were as follows: (1) age < 18 years; (2) patients with other causes of albuminuria, including primary or secondary nephritis, renal or other urological tumors, stones, or trauma-induced persistent proteinuria; (3) recent use of medications that may lower urinary protein excretion, such as angiotensin-converting enzyme inhibitors (ACEI) or angiotensin II receptor blockers (ARB); (4) recent use of lipid-lowering medications; and (5) missing essential study data (TG, HDL-C, or UACR). After screening, 2,323 participants constituted the ultimate cohort (Figure 1).

### 2.2 Data collection

We collected data on the patient’s general characteristics of age, sex, height, weight, waist circumference, the history of drinking, smoking, and blood pressure through a same questionnaire. Blood samples were drawn after a 10-h overnight fast in the morning to measure the following laboratory parameters: urea nitrogen (UN), serum creatinine (Scr), serum uric acid (SUA), fasting blood glucose (FBG) and insulin (INS), glycated hemoglobin (HbA1c), alanine transaminase (ALT), aspartate aminotransferase (AST), free fatty acids (FFA), triglyceride (TG), total cholesterol (TC), low-density lipoprotein cholesterol (LDL-C), high-density lipoprotein cholesterol (HDL-C). Body mass index (BMI) was derived by dividing weight (kg) by height squared (m^2^). Insulin resistance was assessed using the homeostatic model of assessment (HOMA-IR) calculation (fasting insulin (mU/mL) x glucose (mmol/L)/22.5) (17). The non-HDL-C to HDL-C ratio (NHHR) was calculated as (TC (mg/dL) – HDL-C (mg/dL))/HDL-C (mg/dL) (18). A single spot urine sample was collected to measure urinary albumin, urinary creatinine, and calculate the urinary albumin-to-creatinine ratio (UACR). The CKD Epidemiology Collaboration (CKD-EPI) creatinine-based formula was applied for glomerular filtration rate (GFR) estimation (19).

### 2.3 Variable definitions

The Kidney Disease: Improving Global Outcomes (KDIGO) guideline criterion of UACR ≥ 30 mg/g was applied for albuminuria detection. For hypertension, SBP ≥ 140 mmHg, DBP ≥ 90 mmHg, or documented medical history sufficed. Hyperlipidemia was diagnosed as total cholesterol ≥ 5.2 mmol/L or TG ≥ 1.7 mmol/L or HDL-C ≤ 1.0 mmol/L. Obesity was defined as BMI ≥ 28.0 kg/m2. The International Diabetes Federation criteria for metabolic syndrome (MS) required central obesity — WC thresholds of 90 cm for males and 80 cm for females—coupled with any two of: elevated TG (≥1.7 mmol/L); low HDL-C (males: ≤1.03 mmol/L; females: ≤1.29 mmol/L); hyperglycemia (FBG ≥ 5.6 mmol/L or documented T2D); or hypertension (BP ≥ 130/85 mmHg) (20). People who smoked cigarettes at the time were categorized as current smokers, while those who drank alcohol at the time were categorized as current drinkers.

The TG/HDL-C ratio is calculated by dividing the level of TG by the level of HDL-C. Four groups of participants were created based on the TG/HDL-C quartiles: Q1 (TG/HDL-C ≤ 0.80 mmol/L), Q2 (0.80 mmol/L < TG/HDL-C ≤ 1.31 mmol/L), Q3 (1.31 mmol/L < TG/HDL-C ≤ 2.26 mmol/L), and Q4 (TG/HDL-C > 2.26 mmol/L).

### 2.4 Statistical analysis

Variables were reported as medians (interquartile range, IQR) or percentages (%) for continuous and categorical types, respectively. Skewed continuous variables were compared between two groups with the Mann–Whitney *U* test and among multiple groups with the Kruskal–Wallis test. Categorical variables were analyzed with the Chi-squared test. Logistic regression analysis was used to test the association between TG/HDL-C (continuous/quartile) and albuminuria in different models. Furthermore, multicollinearity was assessed for all variables in the regression analysis, and those with variance inflation factor (VIF) > 5 were excluded (Supplementary Table S1). In model 1, no covariates were adjusted. In model 2, age, gender, smoking and drinking status were adjusted. Model 3 was adjusted for age, gender, smoking status, drinking status, hypertension, hyperlipidemia, BMI, HbAlc, and eGFR. And Model 4 added adjustments for HOMA-IR. The possible nonlinear link between TG/HDL-C ratio and albuminuria was assessed using restricted cubic spline (RCS) models fitted for the logistic regression model. When nonlinearity was detected, a piecewise linear relationship was established through segmented regression analysis. To evaluate heterogeneity, subgroup stratification analyses were performed via stratified logistic regression, with the likelihood ratio test subsequently applied to examine subgroup interaction effects. In addition, to assess the mediating roles of blood glucose (FBG) and oxidative stress (serum uric acid [SUA]) in the association between TG/HDL-C and albuminuria in patients with T2D, a mediating effect analysis was performed utilizing the “mediation” package in R, with adjustments made for the factors in Model 4. Receiver operating characteristic (ROC) curves and decision curves (DCA) were used to compare TG/HDL-C with other metabolic markers for albuminuria. All statistical analysis was performed with SPSS (IBM, version 27.0), R (version 4.4.1), and Prism (GraphPad, version 10.1.2). Two-sided *p*-values < 0.05 was deemed statistically significant.

### 2.5 Ethics statement

The study protocol was in accordance with STROBE guidelines (21), and obtained approval from the Ethics Committees of Affiliated Hospital of Medical College Qingdao University and Yuhuan Second People’s Hospital, with written informed consent from all participants.

## 3 Results

### 3.1 Baseline characteristics

As shown in Table 1, a total of 2,323 diabetes patients were enrolled in this study, including 1,545 patients in non-albuminuria group and 778 patients in albuminuria group. Among them, 1,355 (or 58.3% of the total) were male. In the albuminuria group, the male proportion was elevated at 61.1%, along with higher rates of hypertension, obesity, hyperlipidemia, and fatty liver. Compared with non-albuminuria group, DBP, BMI, WC, FBG, FINS, HbAlc, ALT, AST, UN, Scr, UA, TG, TC, FFA, UACR, HOMA-IR and TG/HDL-C were significantly increased in albuminuria group (*p* < 0.05), while e-GFR and HDL-C were decreased (*p* < 0.001). Notably, patients with albuminuria had a markedly higher value of TG/HDL-C (*p* < 0.001).

**Table 1:** Patient demographic and clinical parameters according to UACR.

Variables	Total (*N* = 2,323)	Non-albuminuria (*N* = 1,545)	Albuminuria (*N* = 778)	*P*-value
Age (year)	58 (49–67)	58 (49–67)	58 (48–67)	0.745
Male, *n* (%)	1,355 (58.3%)	879 (56.8%)	476 (61.1%)	0.048*
SBP (mmHg)	78 (70–85)	77 (70–85)	78.5 (70–87)	0.129
DBP (mmHg)	133 (122–146)	132 (120–144)	137 (124–150)	<0.001*
BMI (kg/m^2^)	25.5 (23.4–27.7)	25.3 (23.3–27.4)	25.9 (23.5–28.4)	<0.001*
WC (cm)	92 (85–99)	92 (85–98.15)	93 (86–100)	0.025*
FBG (mmol/L)	7.21 (5.91–9.23)	7 (5.80–8.76)	7.80 (6.15–10.67)	<0.001*
FINS (μIU/ml)	7.87 (4.55–13.45)	7.35 (4.41–12.72)	8.88 (4.87–14.81)	<0.001*
HbAlc (%)	7.9 (6.8–9.6)	7.7 (6.7–9.3)	8.4 (7.1–10.13)	<0.001*
ALT (IU/L)	20 (14–32)	20 (14–31)	21 (14–35)	0.030*
AST (IU/L)	19 (15–25)	18 (15–24)	19 (15–27)	0.009*
UN (mmol/L)	5.6 (4.56–6.8)	5.52 (4.51–6.61)	5.81 (4.67–7.31)	<0.001*
Scr (μmol/L)	58 (47–71)	56 (46–67)	64 (51–81)	<0.001*
e-GFR (ml/min/1.73 m^2^)	103.17 (91.96–113.09)	104.5 (95.23–113.55)	99.8 (80.77–112.19)	<0.001*
UA (μmol/L)	317 (261–386)	308 (256–365)	347.5 (277–432)	<0.001*
TG (mmol/L)	1.45 (0.98–2.23)	1.32 (0.91–2.01)	1.72 (1.18–2.63)	<0.001*
TC (mmol/L)	4.83 (4.03–5.71)	4.76 (4–5.6)	4.97 (4.18–5.89)	<0.001*
HDL-C (mmol/L)	1.11 (0.93–1.32)	1.13 (0.94–1.35)	1.08 (0.90–1.26)	<0.001*
LDL-C (mmol/L)	2.81 (2.18–3.43)	2.8 (2.18–3.4)	2.84 (2.17–3.46)	0.578
UACR (mg/g)	14.62 (6.88–46.85)	8.41 (5.3–14.36)	87.55 (45.97–248.52)	<0.001*
NHHR	3.25 (2.45–4.29)	3.13 (2.35–4.05)	3.63 (2.72–4.85)	<0.001*
TG/HDL-C	1.31 (0.8–2.26)	1.19 (0.74–2)	1.65 (0.95–2.74)	<0.001*
HOMA-IR	2.68 (1.36–4.82)	2.47 (1.28–4.34)	3.39 (1.65–5.97)	<0.001*
Current smokers, *n* (%)	724 (31.1%)	467 (30.2%)	257 (33%)	0.168
Current drinkers, *n* (%)	745 (32%)	489 (31.6%)	256 (32.9%)	0.541
Hypertension, *n* (%)	1,418 (61%)	869 (56.2%)	549 (70.5%)	<0.001*
Hyperlipidemia, *n* (%)	1,671 (71.9%)	1,063 (68.8%)	608 (78.1%)	<0.001*
Metabolic syndrome, *n* (%)	1,428 (61.4%)	947 (61.2%)	481 (61.8%)	0.804
Obesity, *n* (%)	543 (23.3%)	317 (20.5%)	226 (29%)	<0.001*
Fatty liver, *n* (%)	1,247 (53.6%)	799 (51.7%)	448 (57.5%)	0.007*

Data are presented as counts (%) or medians (interquartile ranges).

SBP, systolic blood pressure; DBP, diastolic blood pressure; BMI, body mass index; WC, waist circumference; FBG, fasting blood glucose; FINS, fasting insulin level; HbA1c, glycated hemoglobin; ALT, alanine transaminase; AST, aspartate aminotransferase; UN, urea nitrogen; Scr, serum creatinine; e-GFR, estimated glomerular filtration rate; UA, uric acid; TG, triglycerides; TC, total cholesterol; HDL-C, high-density lipoprotein cholesterol; LDL-C, low-density lipoprotein cholesterol; FFA: free fatty acid; UACR, urinary albumin-to-creatinine ratio; HOMA-IR, homeostatic model assessment of insulin resistance; TG/HDL-C, triglycerides/high-density lipoprotein cholesterol ratio.

*p < 0.05.

### 3.2 Clinical characteristics of TG/HDL-C quartile stratification

**Figure 2:**
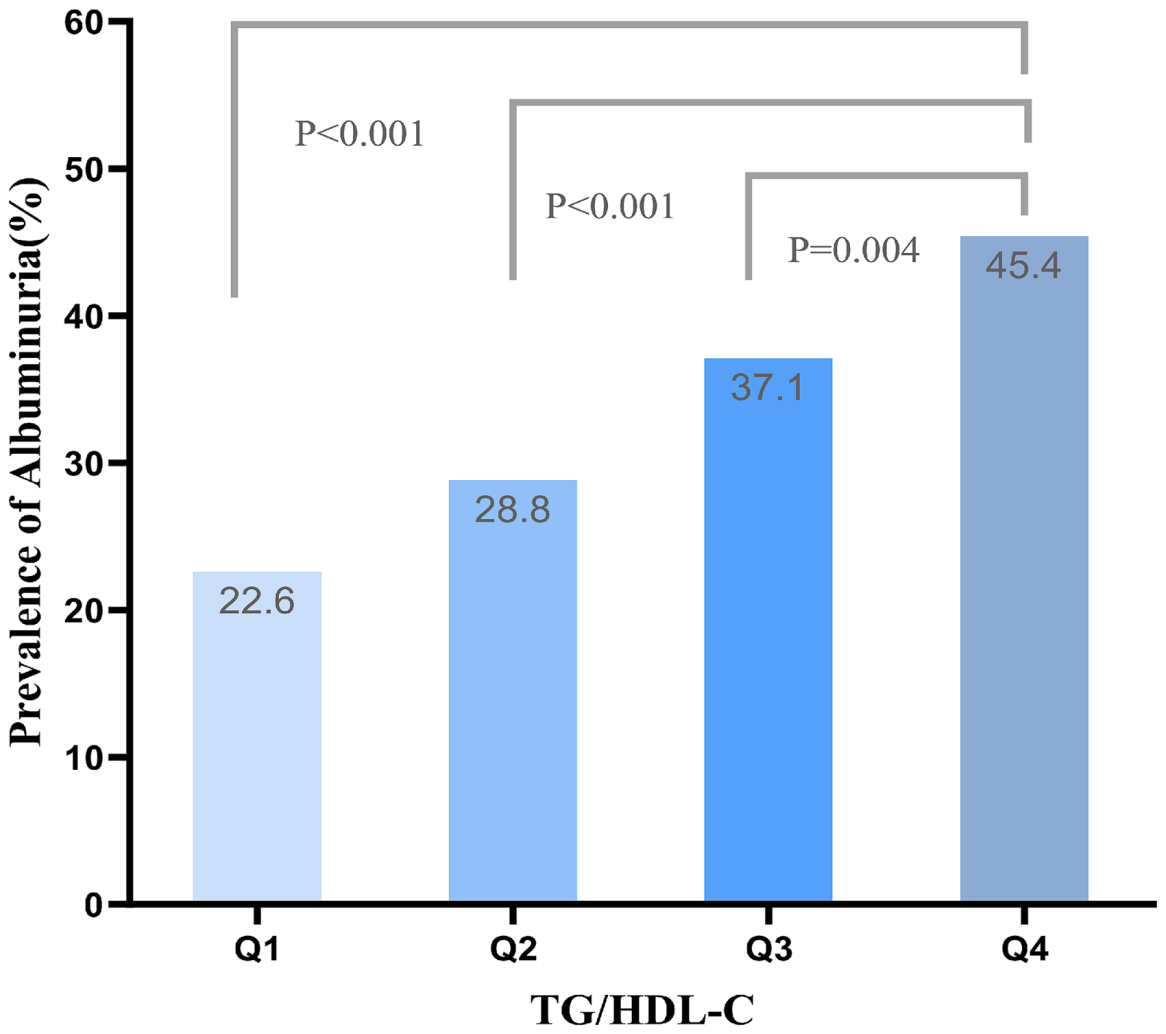
The prevalence of albuminuria in different groups of TG/HDL-C ratio.

Based on the TG/HDL-C level of the subjects, they were categorized into four quartiles: Q1 (TG/HDL-C ≤ 0.80 mmol/L), Q2 (0.80 mmol/L < TG/HDL-C ≤ 1.31 mmol/L), Q3 (1.31 mmol/L < TG/HDL-C ≤ 2.26 mmol/L), and Q4 (TG/HDL-C > 2.26 mmol/L) (Table 2). Participants with a greater TG/HDL-C ratio were markedly younger, having a higher proportion of male, higher prevalence of hyperlipidemia, obesity and fatty liver, higher rates of smoking and alcohol consumption, higher SBP, BMI, WC, FBG, FINS, HbAlc, ALT, AST, Scr, e-GFR, UA, TG, TC, LDL-C, FFA and HOMA-IR, while lower HDL-C concentrations (*p* < 0.05). Moreover, the UACR level (9.47 vs. 13.26 vs. 15.38 vs. 24.75, *p* < 0.001) and the number of people with albuminuria (22.6% vs. 28.8% vs. 37.1% vs. 45.4%, *p* < 0.001) showed a significant upward trend across TG/HDL-C quartiles. As depicted in Figure 2, the prevalence of albuminuria notably escalates in Q4 compared to Q1, Q2, and Q3 (*p* < 0.05).

**Table 2:** Clinical characteristics based on TG/HDL-C quartile stratification.

Variables	Q1 (≤0.8) *N* = 582	Q2 (0.8–1.31) *N* = 583	Q3 (1.31–2.26) *N* = 581	Q4 (>2.26) *N* = 577	*P*-value
Age (year)	62 (53–70)	59 (52–68)	58 (48–67)	52 (43–60)	<0.001*
Male, *n* (%)	280 (48.1%)	314 (53.8%)	340 (58.5%)	421 (72.9%)	<0.001*
SBP (mmHg)	76 (68–84)	77 (70–85)	78 (70–85.5)	80 (72–87)	<0.001*
DBP (mmHg)	132 (120–145)	135 (123–147)	133 (122–146)	134 (122–146)	0.24
BMI (kg/m^2^)	23.8 (21.98–25.9)	25.2 (23.4–27.4)	26 (23.8–28.2)	26.4 (24.85–28.85)	<0.001*
WC (cm)	89 (82–95)	91 (85–98.3)	93 (86–100)	95 (85–100.1)	<0.001*
FBG (mmol/L)	6.48 (5.26–8.18)	6.86 (5.8–8.77)	7.32 (6.04–9.29)	8.21 (6.73–11.08)	<0.001*
FINS (μIU/mL)	4.96 (2.78–8.9)	7.27 (4.2–12.57)	9.11 (5.61–14.7)	10.65 (6.56–16.79)	<0.001*
HbAlc (%)	7.7 (6.6–9.33)	7.8 (6.8–9.4)	8 (6.9–9.45)	8.3 (7.1–10)	<0.001*
ALT (IU/L)	17 (13–24)	19 (13–28)	22 (15–34)	27 (17–46)	<0.001*
AST (IU/L)	18 (14–22)	18 (14–23)	19 (15–27)	20 (15–29)	<0.001*
UN (mmol/L)	5.69 (4.7–6.8)	5.65 (4.64–6.93)	5.49 (4.49–6.82)	5.53 (4.5–6.67)	0.241
Scr (μmol/L)	54 (45–65)	57 (47–69)	61 (49–74)	63 (51–75.5)	<0.001*
e-GFR (ml/min/1.73 m^2^)	102.1 (93.48–110.65)	101.74 (91.52–111.51)	102.4 (90.01–112.23)	107.01 (94.77–116.62)	<0.001*
UACR (mg/g)	9.47 (5.74–27.4)	13.26 (6.61–35.59)	15.38 (7.07–47.1)	24.75 (9.72–93.26)	<0.001*
UA (μmol/L)	274.5 (228–328.25)	312 (261–367)	332 (277.5–394)	369 (302–443)	<0.001*
TG (mmol/L)	0.8 (0.65–0.93)	1.21 (1.03–1.40)	1.74 (1.5–2.04)	3.15 (2.55–4.5)	<0.001*
TC (mmol/L)	4.61 (3.75–5.36)	4.71 (3.98–5.55)	4.82 (4.07–5.72)	5.23 (4.36–6.18)	<0.001*
HDL-C (mmol/L)	1.41 (1.23–1.63)	1.17 (1.02–1.32)	1.03 (0.91–1.17)	0.9 (0.77–1.04)	<0.001*
LDL-C (mmol/L)	2.61 (1.92–3.21)	2.83 (2.25–3.43)	2.95 (2.37–3.63)	2.77 (2.18–3.43)	<0.001*
NHHR	2.23 (1.70–2.74)	2.97 (2.43–3.58)	3.67 (3.06–4.32)	4.82 (3.91–5.76)	<0.001*
HOMA-IR	1.45 (0.78–2.89)	2.49 (1.23–4.2)	3.11 (1.79–5.13)	4.04 (2.42–6.7)	<0.001*
Current smokers, *n* (%)	131 (22.5%)	155 (26.5%)	195 (33.5%)	243 (42.1%)	<0.001*
Current drinkers, *n* (%)	143 (24.5%)	160 (27.4%)	201 (34.5%)	241 (41.7%)	<0.001*
Hypertension, *n* (%)	355 (60.9%)	349 (59.8%)	350 (60.2%)	364 (63%)	0.68
Hyperlipidemia, *n* (%)	215 (36.9%)	344 (59.8%)	535 (92%)	577 (100%)	<0.001*
Metabolic syndrome, *n* (%)	370 (63.5%)	375 (64.3%)	346 (59.5%)	337 (58.4%)	0.099
Obesity, *n* (%)	69 (11.8%)	125 (21.4%)	151 (25.9%)	198 (34.3%)	<0.001*
Fatty liver, *n* (%)	220 (37.8%)	289 (49.5%)	354 (60.9%)	384 (66.5%)	<0.001*
Albuminuria, *n* (%)	132 (22.6%)	168 (28.8%)	216 (37.1%)	262 (45.4%)	<0.001*

Data are presented as counts (%) or medians (interquartile ranges).

TG/HDL-C, triglycerides/high-density lipoprotein cholesterol ratio; SBP, systolic blood pressure; DBP, diastolic blood pressure; BMI, body mass index; WC, waist circumference; FBG, fasting blood glucose; FINS, fasting insulin level; HbA1c, glycated hemoglobin; ALT, alanine transaminase; AST, aspartate aminotransferase; UN, urea nitrogen; Scr, serum creatinine; e-GFR, estimated glomerular filtration rate; UACR, urinary albumin-to-creatinine ratio; UA, uric acid; TG, triglycerides; TC, total cholesterol; HDL-C, high-density lipoprotein cholesterol; LDL-C, low-density lipoprotein cholesterol; FFA, free fatty acid; HOMA-IR, homeostatic model assessment of insulin resistance.

*p < 0.05 vs. Q1 cohort.

### 3.3 Association of the clinical variables with albuminuria in individuals with diabetes

**Figure 3:**
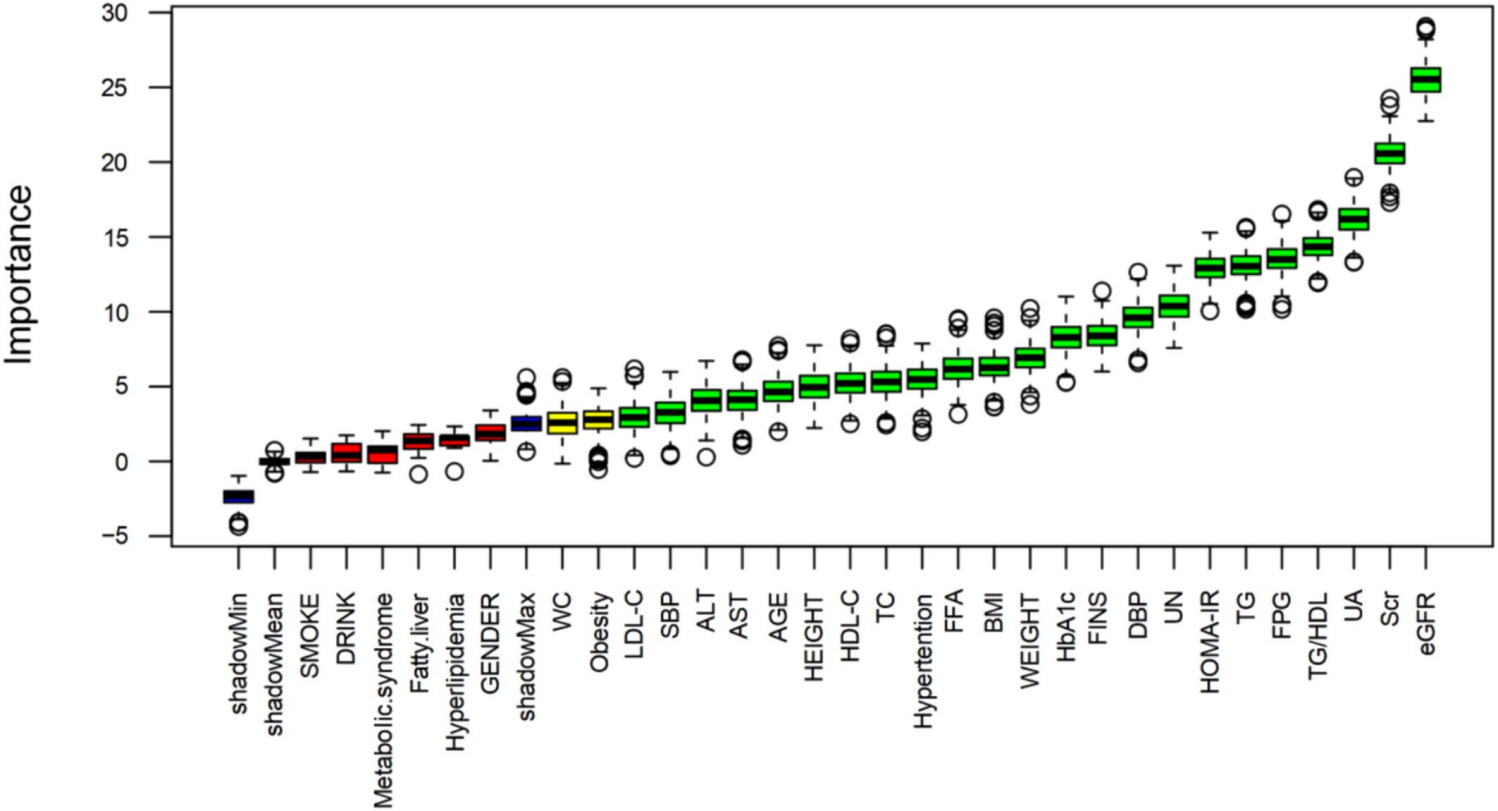
Feature selection with Boruta. The blue plot shows minimum, average, and max shadow score. Variables having box plot in green are important, in yellow as tentative, and in red as rejected.

To identify the predictors of albuminuria, univariate logistic regression analysis was performed. The results demonstrated that albuminuria exhibited positive correlations with gender, DBP, BMI, WC, FBG, FINS, HbA1c, ALT, AST, UN, Scr, UA, FFA, TC, TG, TG/HDL-C, HOMA-IR, along with the prevalence of hyperlipidemia, hypertension, obesity, and fatty liver while showing negative correlations with HDL-C and e-GFR among all participants (*p* < 0.05) (Table 3). Of note, TG/HDL-C was one of the valuable lipid indicators associated with albuminuria. Moreover, through the findings from feature screening with Boruta’s algorithm in Figure 3, TG/HDL-C was one of the variables most closely linked with albuminuria. Therefore, this study mainly explored correlation of TG/HDL-C with albuminuria occurrence in T2D patients.

**Table 3:** Univariate logistic regression analysis with UACR as the dependent variable.

Risk factor	OR (95% CI)	*p*-value
Gender (male, %)	1.194 (1.002–1.424)	0.048*
DBP (mmHg)	1.016 (1.011–1.021)	<0.001*
BMI (kg/m^2^)	1.057 (1.033–1.081)	<0.001*
WC (cm)	1.010 (1.002–1.018)	0.014*
FBG (mmol/L)	1.113 (1.084–1.143)	<0.001*
FINS (μIU/mL)	1.021 (1.013–1.030)	<0.001*
HbAlc (%)	1.151 (1.105–1.198)	<0.001*
ALT (IU/L)	1.003 (1.001–1.006)	0.015*
AST (IU/L)	1.004 (1.001–1.008)	0.016*
UN (mmol/L)	1.148 (1.104–1.194)	<0.001*
Scr (μmol/L)	1.020 (1.017–1.025)	< 0.001*
e-GFR (ml/min/1.73 m^2^)	0.980 (0.976–0.984)	<0.001*
UA (μmol/L)	1.004 (1.003–1.005)	<0.001*
TG (mmol/L)	1.134 (1.084–1.187)	<0.001*
TC (mmol/L)	1.170 (1.098–1.247)	<0.001*
HDL-C (mmol/L)	0.440 (0.329–0.589)	<0.001*
NHHR	1.248 (1.178–1.321)	<0.001*
TG/HDL-C	1.077 (1.043–1.112)	<0.001*
HOMA-IR	1.087 (1.063–1.112)	< 0.001*
Hypertension	1.865 (1.551–2.242)	<0.001*
Hyperlipidemia	1.622 (1.326–1.983)	<0.001*
Obesity	1.586 (1.301–1.933)	<0.001*
Fatty liver	1.268 (1.065–1.508)	0.007*

UACR, urinary albumin-to-creatinine ratio; DBP, diastolic blood pressure; BMI, body mass index; WC, waist circumference; FBG, fasting blood glucose; FINS, fasting insulin level; HbA1c, glycated hemoglobin; ALT, alanine transaminase; AST, aspartate aminotransferase; UN, urea nitrogen; Scr, serum creatinine; e-GFR, estimated glomerular filtration rate; UA, uric acid; FFA: free fatty acid; TG, triglycerides; TC, total cholesterol; HDL-C, high-density lipoprotein cholesterol; TG/HDL-C, triglycerides/high-density lipoprotein cholesterol ratio; HOMA-IR, homeostatic model assessment of insulin resistance.

*p < 0.05.

### 3.4 Relationship of TG/HDL-C with albuminuria prevalence

We utilized multivariate logistic regression analysis to further explore the correlation between TG/HDL-C and albuminuria (Table 4). After progressively adjusting for all potential confounders, elevated TG/HDL-C were found to be significantly associated with a higher prevalence of albuminuria in all regression models. When TG/HDL-C ratio was transformed into a categorical variable, we observed that the ORs (95% CI) of albuminuria across the highest to the lowest quartiles were 2.141 (1.533–2.990), 1.742 (1.272–2.385), 1.222 (0.915–1.630), respectively in model4 (trend *p*-value < 0.001).

**Table 4:** The association between TG/HDL-C and albuminuria.

Variable	Model 1	*P*-value	Model 2	*P*-value	Model 3	*P*-value	Model 4	*P*-value
	OR (95% CI)		OR (95% CI)		OR (95% CI)		OR (95% CI)	
TG/HDL-C	1.077 (1.043–1.112)	<0.001*	1.078 (1.043–1.115)	<0.001*	1.046 (1.014–1.080)	0.005*	1.039 (1.008–1.071)	0.012*
Quartile 1	–	–	–	–	–	–	–	–
Quartile 2	1.380 (1.060–1.797)	0.017*	1.393 (1.069–1.815)	0.014*	1.248 (0.937–1.663)	0.130	1.222 (0.915–1.630)	0.174
Quartile 3	2.017 (1.561–2.608)	<0.001*	2.074 (1.600–2.688)	<0.001*	1.770 (1.294–2.420)	<0.001*	1.742 (1.272–2.385)	<0.001*
Quartile 4	2.835 (2.200–3.655)	<0.001*	3.023 (2.308–3.943)	<0.001*	2.299 (1.651–3.201)	<0.001*	2.141 (1.533–2.990)	<0.001*
*P* for trend	<0.001*		<0.001*		<0.001*		<0.001*	

OR, odds ratio; CI, confidence interval.

Model 1: no covariates were adjusted.

Model 2: adjustments for age, gender, smoking status and drinking status.

Model 3: adjustments for Model 2 variables plus hypertension, hyperlipidemia, BMI, HbAlc, and e-GFR.

Model 4: additionally adjusted for HOMA-IR.

*P < 0.05.

### 3.5 Non-linear connections between TG/HDL-C and albuminuria in individuals with diabetes

**Figure 4:**
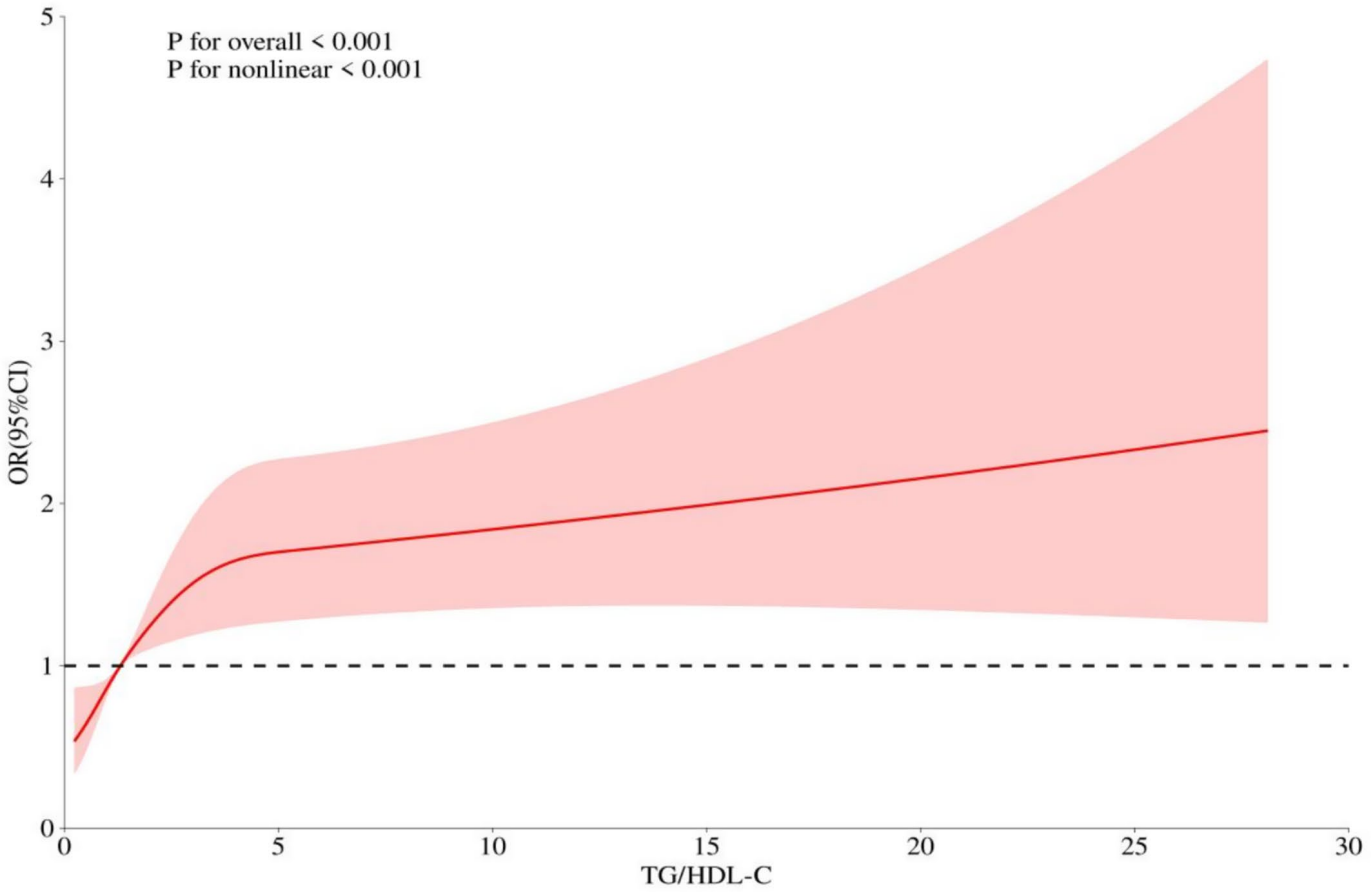
Restricted cubic spline (RCS) analysis of the relationships between TG/HDL-C and albuminuria. The solid line represents the OR, with the shaded area representing the 95% CI. Adjustments were made to the model for age, gender, smoking status, drinking status, hyperlipidemia, hypertension, e-GFR, BMI, HbAlc and HOMA-IR.

To explore the relationship of TG/HDL-C with proteinuria in diabetic patients, we employed the RCS curve model. The results demonstrated an obvious nonlinear relationship between TG/HDL-C and albuminuria risk, independent of confounders (*P* for non-linear < 0.001) (Figure 4).

Furthermore, threshold effect analysis determined an inflection point of 2.08 through two-piecewise linear regression (*P* for likelihood test < 0.001). Below this threshold, TG/HDL-C exhibited a significant positive correlation with albuminuria, with an odds ratio of 1.55 (95% CI, 1.21–1.98). Conversely, beyond the turning point, the association was no longer statistically evident (*p* > 0.05) (Table 5).

**Table 5:** The result of the two-piecewise linear regression model.

Outcome	Effect	*P*
Model 1 Fitting model by standard linear regression	1.04 (1.01–1.08)	**0.006**
Model 2 Fitting model by two-piecewise linear regression		
Inflection point	2.08	
<2.08	1.55 (1.21–1.98)	**<0.001**
≥2.08	1.02 (0.99–1.04)	0.254
*P* for likelihood test		**<0.001**

Bold values indicate *P* < 0.05.

### 3.6 Subgroup analysis and mediation analyses

**Figure 5:**
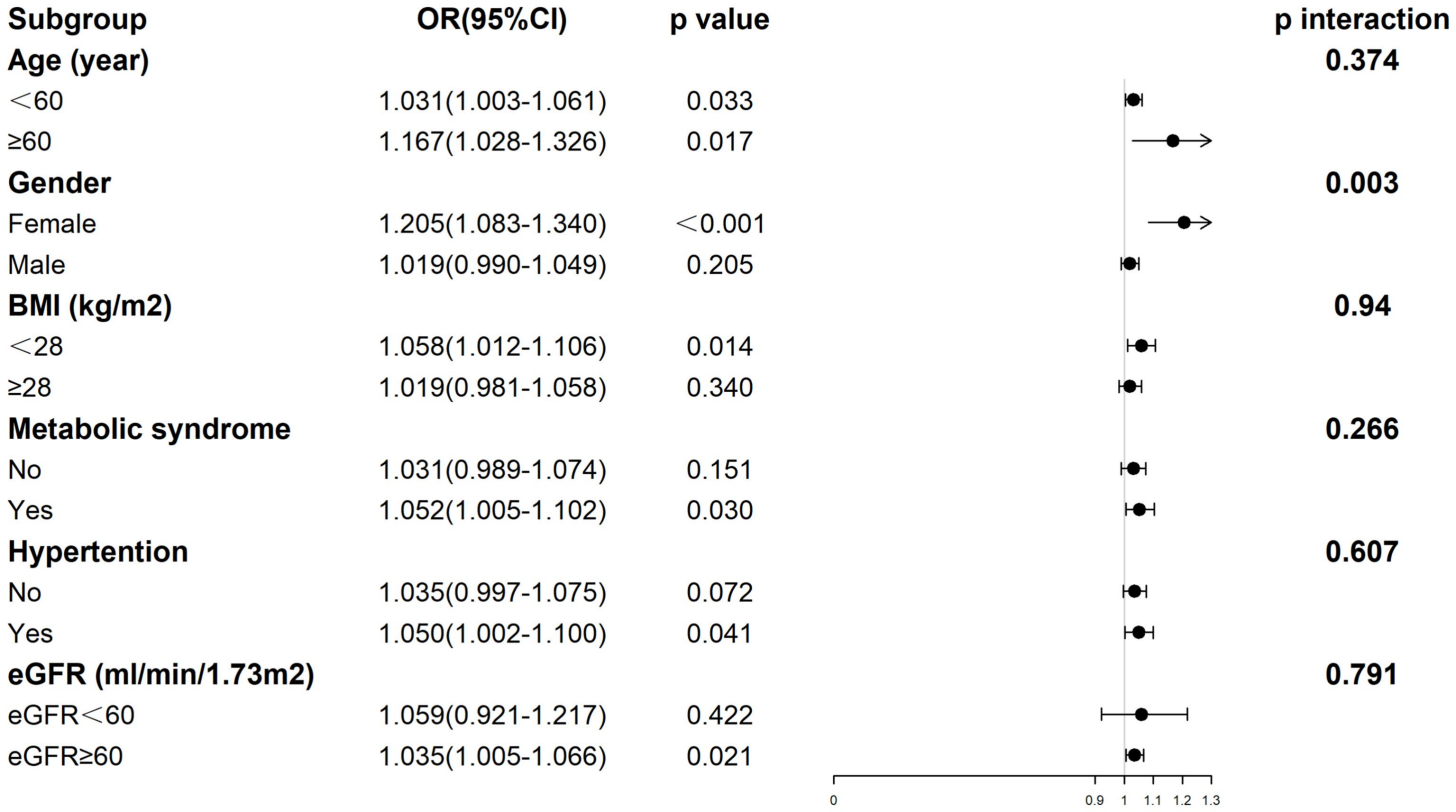
Subgroup analyses of the association between TG/HDL-C and albuminuria.

In Figure 5, subgroup analyses were conducted to examine potential effect modification. The interaction test suggested that the relationship between TG/HDL-C and albuminuria was influenced by gender stratification (*p* for interaction = 0.003), with a relatively stronger association observed among female participants [OR (95% CI): 1.205 (1.083–1.340)]. The results were generally consistent across other factors, including age, BMI, hypertension, hyperlipidemia, metabolic syndrome, and eGFR (all *p* for interaction > 0.05).

**Figure 6:**
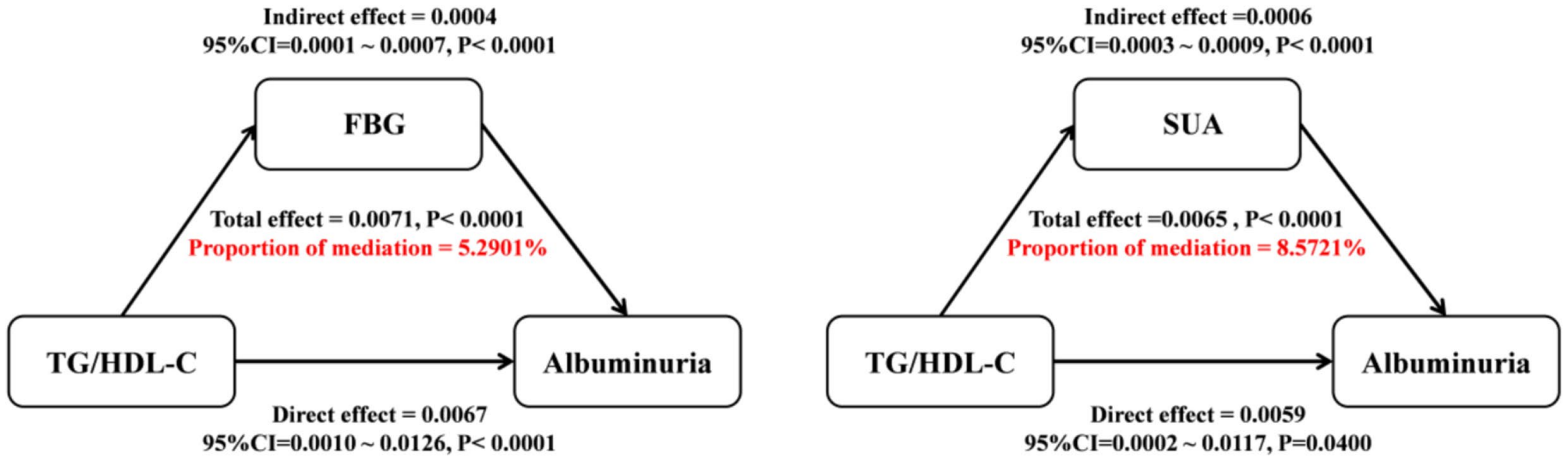
Mediating effects of FBG, SUA on the association between TG/HDL-C and albuminuria risk. The model adjusted for age, gender, smoking status, drinking status, hyperlipidemia, hypertension, e-GFR, BMI, HbAlc and HOMA-IR. Direct effect: association independent of mediators. Indirect effect: association via FBG (5.29%) and SUA (8.57%).

Mediation analysis revealed that, following multivariate adjustment, TG/HDL-C was associated with albuminuria through pathways that may involve blood glucose and oxidative stress (SUA). Specifically, FBG and SUA were estimated to account for 5.29 and 8.57% of the total association, respectively (*p* < 0.01) (Figure 6).

### 3.7 Predictive value of TG/HDL-C for albuminuria risk in T2D patients

**Figure 7:**
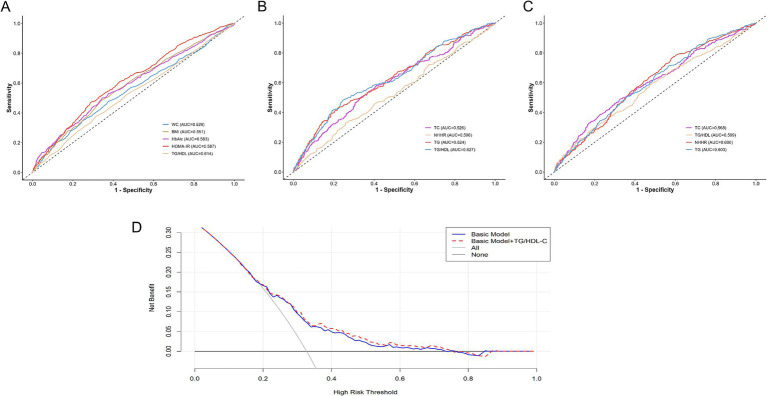
**(A)** Receiver operating characteristic (ROC) curve of the TG/HDL-C as predictor of albuminuria. **(B)** Predictive utility test of lipid parameters for albuminuria risk in female with T2D. **(C)** Predictive utility test of lipid parameters for albuminuria risk in man with T2D. **(D)** Decision curve analysis (DCA).

The ROC curve analysis was used to exploratorily compare the relative discriminative ability of TG/HDL-C with other metabolic markers for albuminuria (Figure 7). The area under the curve (AUC) for TG/HDL-C was 0.614 (95% CI, 0.590–0.638), with 71.0% sensitivity and 47.0% specificity. The AUC of HOMA-IR, HbA1c, BMI, and WC was 0.587 (0.562–0.612), 0.583 (0.559–0.608), 0.551 (0.526–0.576), and 0.529 (0.503–0.554), respectively. Furthermore, among the various lipid indicators for albuminuria in T2D patients, the TG/HDL-C ratio showed relatively better discrimination in female patients. In addition, potential net benefit was assessed through decision curve analysis (DCA). The model with TG/HDL-C added showed a modestly increased net benefit.

## 4 Discussion

An in-depth analysis of 2,323 participants revealed that the TG/HDL-C ratio was strongly linked to a greater risk of albuminuria, even after controlling for potential confounders. Notably, this association followed a non-linear pattern, with a distinct inflection point at a TG/HDL-C value of 2.08. Furthermore, gender significantly modified this relationship, with stronger associations observed among female patients with type 2 diabetes. Additionally, our findings suggested that TG/HDL-C may indirectly influence the development of albuminuria by modulating FBG and SUA levels.

Various investigations have established robust connections between dyslipidemia and albuminuria (22, 23). Based on previous findings, albuminuria risk was shown to be higher in patients with lower serum HDL-C levels, higher TC and TG concentrations (22, 24). Additionally, in the study, Yu et al. demonstrated that the lipid accumulation product (LAP) could serve as a potential biomarker for CKD, often accompanied by albuminuria (25). A cross-sectional study involving 9,872 participants in the United States revealed a positive correlation between the triglyceride-glucose (Ty-G) index and the risk of albuminuria, independent of insulin resistance (26). Another cohort study in the Chinese community highlighted a strong association between the Chinese visceral adiposity index (CVAI) and elevated UACR, particularly among individuals with hyperglycemia or hypertension (27). In summary, an increasing number of studies are now advocating for the use of novel composite lipid indices to predict albuminuria.

This study confirmed earlier findings that the TG/HDL-C ratio positively correlated with proteinuria in T2D patients. A cross-sectional study involving 32,877 participants from eight regional centers in China identified TG/HDL-C as an independent positive predictor of microalbuminuria (13). After adjusting for confounding variables, Tam et al. observed that rising TG/HDL-C coincided with increased risk of diabetic nephropathy and higher urine albumin excretion in a large Turkish population (28). Moreover, our findings revealed a significant nonlinear relationship, with a fold point identified at 2.08. The results suggested that below the TG/HDL-C threshold of 2.08, each unit increase was associated with a 55% higher risk of albuminuria. This threshold may serve as an epidemiological reference for risk stratification, though its applicability to individual-level clinical decision-making requires prospective validation. In exploratory ROC comparisons, TG/HDL-C exhibited relatively higher discrimination for albuminuria compared to traditional obesity and insulin resistance-related markers. Although the absolute AUC (0.614) indicates limited standalone diagnostic utility—consistent with the multifactorial nature of diabetic kidney disease—TG/HDL-C nonetheless outperformed conventional metabolic indicators in this cohort. Furthermore, the relative advantage observed in female patients may reflect sex-specific lipid metabolism patterns and warrants further investigation. These findings support the potential value of TG/HDL-C as a contributory component in multi-marker risk assessment frameworks.

Mediation analysis indicated that FBG and SUA may partially mediate the relationship between the TG/HDL-C ratio and albuminuria in patients with T2D. As a key marker of insulin resistance, an elevated TG/HDL-C ratio exacerbates pancreatic *β*-cell dysfunction and glucolipotoxicity (29), leading to increased FBG levels. Oxidative stress and advanced glycation end products (AGEs), which arise from persistent hyperglycemia, mediate damage to the glomerular filtration barrier (30). Additionally, the elevated TG/HDL-C ratio inhibits renal uric acid excretion and enhances its production. SUA, acting as an oxidant, further exacerbates renal tubular injury by activating the NLRP3 inflammasome and inducing endothelial dysfunction (31). However, causality cannot be inferred from this cross-sectional design. The observed pathways may reflect concurrent correlations, reverse causality, or unmeasured confounding, and should be interpreted as hypothesis-generating. Validation in prospective or interventional studies is warranted.

Our subgroup analysis showed that the TG/HDL-C–albuminuria association varied by sex, with the interaction test suggesting gender as a potential effect modifier (*p* = 0.003). This observation is consistent with findings from multiple cross-regional studies. Data from a representative Chinese cohort indicated that the relationship between TG/HDL-C and UACR was prevalent among females across all subgroups, including prehypertension, normal/overweight BMI, prediabetes, and all age groups. In contrast, this association in males was only evident within the overweight subgroup (14). Additionally, a study in South Korea demonstrated that the TG/HDL-C association with UACR diminished among men after adjusting for systolic blood pressure (*p* = 0.24), while it remained significant in women (*p* = 0.04) (13). Potential mechanisms may include the loss of estrogen’s protective effects and variations in metabolic phenotypes (32, 33). Estrogen protects the kidneys through the GPR30 receptor (34), but dyslipidemia and central obesity in women work synergistically to amplify inflammation in renal tissues, thereby accelerating damage to the glomerular filtration barrier via tumor necrosis factor-alpha (TNF-*α*) and interleukin-6 (IL-6) (32, 35), with this effect being more pronounced after menopause. Moreover, metabolic dysregulation of subcutaneous fat in women increases the release of free fatty acids, worsening insulin resistance and promoting lipid accumulation in the kidneys (36). In contrast, albuminuria in men is mainly driven by conventional high-risk factors such as hypertension and smoking, potentially masking the independent contribution of dyslipidemia (37). Despite this biological plausibility, these sex-specific findings remain hypothesis-generating and require confirmation in adequately powered studies.

The mechanisms underlying the association between the TG/HDL-C ratio and albuminuria in diabetic patients may be linked to factors such as insulin resistance, renal lipotoxicity, and chronic low-grade inflammation. On one hand, as a reliable surrogate marker of insulin resistance (38), higher TG/HDL-C reflects hyperinsulinemic status, which directly activates the renin-angiotensin-aldosterone system (RAAS), triggering glomerular hyperfiltration, increased pressure, and enhanced sodium reabsorption (39). This cascade sequentially induces endothelial dysfunction, increases vascular permeability, and ultimately promotes albuminuria (40). Additionally, dysregulated lipid metabolism leads to lipid deposition in the mesangial area, stimulating the secretion of pro-inflammatory cytokines, directly disrupting podocyte cytoskeletal integrity, impairing mitochondrial function, and depleting ATP, all of which compromise the selectivity of the filtration barrier (41, 42). Concurrently, reduced HDL-C levels impair its reverse cholesterol transport and anti-inflammatory, antioxidant functions, exacerbating glomerular lipid peroxidation and sclerosis (43). On the other hand, elevated TG/HDL-C, coupled with dysfunctional adipose tissue, drives macrophage polarization toward the pro-inflammatory M1 phenotype, releasing inflammatory cytokines such as TNF-αand IL-6, thus inducing a chronic low-grade inflammatory state that contributes to renal damage (44–46). In turn, renal insufficiency exacerbates insulin resistance in adipose tissue (47) and systemic lipid metabolism disorders, creating a vicious cycle.

This study has several strengths. First, we utilized data from two centers, expanding the sample size and enriching the diversity of the data. Second, we adjusted for confounding variables and performed further stratified analyses to improve the reliability of the results. Nonetheless, this study has certain limitations. First, the study’s cross-sectional structure restricts causal conclusions about TG/HDL-C effects on albuminuria. Second, the ethnic specificity of our Chinese sample restricts the external validity of our conclusions. Third, a single spot urine sample may not fully represent an individual’s albuminuric burden due to the fluctuating nature of urinary albumin excretion over time, thereby increasing the risk of misclassification. Fourth, several important confounders remain unmeasured in our dataset, including diabetes duration, hypertension duration, diabetes treatment intensity (e.g., insulin dosage, number of oral hypoglycemic agents), physical activity level, and detailed dietary patterns. The absence of these variables may result in residual confounding. For instance, longer diabetes duration and more intensive treatment may reflect worse metabolic control and higher albuminuria risk, potentially exaggerating the observed TG/HDL-C association. Therefore, future prospective studies incorporating repeated urine measurements, comprehensive data collection, large samples, and multiple centers are warranted to validate these findings.

## 5 Conclusion

In conclusion, elevated TG/HDL-C increased albuminuria risk, particularly among females. Avoiding excessive elevation of TG/HDL-C may be a potential target for albuminuria prevention in patients with T2D. And further prospective studies are needed to explore the underlying causal relationship.

## Data Availability

The raw data supporting the conclusions of this article will be made available by the authors, without undue reservation.
