# Supplementary material for: Association between triglyceride-to-high-density lipoprotein cholesterol ratio and albuminuria in patients with type 2 diabetes: a cross-sectional study
Source: Front Nutr. 2026 Jul 1;13:1810515. doi: 10.3389/fnut.2026.1810515 (PMC13368516; doi:10.3389/fnut.2026.1810515)
Supplement: Supplementary file 1 [file Table_1.docx]

Table S1 .Multicollinearity assessment of exposure variables with other covariates.

| Variable | VIF value |
| --- | --- |
| Gender | 1.710 |
| Age(year) | 1.978 |
| Current smokers (%) | 1.656 |
| Current drinkers (%) | 1.667 |
| Hypertention | 1.143 |
| Hyperlipidemia | 1.094 |
| e-GFR(ml/min/1.73 m2) | 1.717 |
| BMI (kg/m^2^) | 1.223 |
| HbAlc(%) | 1.080 |
| HOMA-IR | 1.181 |
| TG/HDL-C | 1.092 |

Note: The variables with VIF>5 will be regarded as collinear variables and cannot be included in the multiple regression model.

Abbreviation: VIF, variance inflation factor; e-GFR, estimated glomerular filtration rate; BMI, body mass index; HbA1c, glycated hemoglobin;HOMA-IR, homeostatic model assessment of insulin resistance;TG/HDL-C, triglycerides/high-density lipoprotein cholesterol ratio.
